# PD-1 blockade reverses viral infection-induced loss of anti-tumor CD8+ T cell responses

**DOI:** 10.1186/2051-1426-1-S1-P156

**Published:** 2013-11-07

**Authors:** Erica Huelsmann, Joseph Broucek, Andrew Lacek, Eugene Lusciks, Johnson Saba, Arman Nabatiyan, Andrew Zloza

**Affiliations:** 1 Department of Immunology/Microbiology, Rush University Medical Center, Chicago, IL, USA; 2 Department of Internal Medicine, Rush University Medical Center, Chicago, IL, USA

## Background

Emerging epidemiologic studies describe an increased prevalence of tumors in patients with non-oncogenic chronic viral disease (e.g., non-AIDS-defining cancers in HIV and non-hepatic cancers in HCV). However, there is a lack of basic understanding by what mechanism infections result in increased unrelated cancer formation (i.e., of cancers in tissues not associated with the infection), and what role immunotherapy may have in the rescue of immune responses under this condition. We hypothesized that viral infections establish an immunological environment that leads to the preferential loss of anti-tumor (i.e., anti-self antigen) CD8+ T cells, resulting in the inability of the host to avert tumor growth, and further that immunotherapy can reverse this detrimental loss.

## Methods

We used a well-characterized mouse melanoma (B16-F10) model and the well-accepted acute infection model of influenza (PR/8). To follow anti-self and non-self CD8+ T cell responses, pmel (specific against melanocyte gp10025-33; expressing Thy1.1) and OT-1 (specific against non-self antigen OVA257-264; expressing Ly5.1) CD8+ T cells were adoptively transferred into B6 (Thy1.1- Ly5.1-) mice. B6 mice were infected with influenza (80,000 EID50) via intranasal (i.n.) injection three days prior to or on the same day as B16 melanoma challenge (120,000 cells via intradermal injection). Some mice received PD-1 or control IgG injection (125µg; i.p.) on days -6, -2, and 0. Tumor area (width*length) was measured every two days until host death and CD8+ T cells were detected by flow cytometry.

## Results

Influenza infection three days prior to tumor challenge (but not concomitantly on day 0) significantly worsened the natural anti-tumor response (specifically, decreased survival from 35 days without to 23 days with infection; P<0.01). Further, influenza infection resulted in the preferential loss of tumor antigen-specific (pmel) but not non-self (OT-I) CD8+ T cells (up to 90%; P<0.001) from the tumor and lymphoid tissues. Importantly, PD-1 blockage reversed the effects of influenza infection on anti-tumor responses and anti-tumor CD8+ T cell loss.

## Conclusions

To our knowledge we are the first to report that anti-tumor CD8+ T cell responses are preferentially lost in the context of unrelated, non-oncogenic, viral infection. These studies may answer the long-standing questions of why certain individuals are more susceptible to tumor formation and less responsive to cancer therapies. Future efforts will test whether these findings are universally applicable to other infections and cancers and are expected to have important clinical cancer immunotherapy implications.

